# Preserving a Space for Science in an Age of Democracy

**DOI:** 10.1371/journal.pbio.1000274

**Published:** 2010-01-05

**Authors:** Harry Collins

**Affiliations:** Centre for the Study of Knowledge Expertise and Science (KES), Cardiff University, Cardiff, United Kingdom

## Abstract

How should scientific advice be incorporated into the political decision-making process? Harry Collins explores this question in his review of *The Paradox of Scientific Authority*.

How should scientific advice be incorporated into the political decisionmaking process? Even experts can't keep up with the torrent of studies published in their own field, and, supposedly, scientific issues—from climate change to biodiversity loss—have obvious political components. How is advice treated in an age when experts are increasingly viewed with suspicion and distrust?

*The Paradox of Scientific Authority: The Role of Scientific Advice in Democracies*, by Wiebe E Bijker, Roland Bal, and Ruud Hendriks, reveals the political decision-making in a study of the inner workings of the “Gezondheidsraad,” a scientific advisory body to the Dutch government. Dutch officials invited Bijker et al. to observe the process and write their account, which was eventually presented to Queen Beatrix. So, it is one of those instances—these days growing in number—where the once-reviled sociologists are being asked to help the powerful understand the relationship between policy and science. The authors are well aware of the problems posed for their objectivity—both actual and perceived—by this dangerous shift from outsider to insider.

The paradox referred to in the title has its roots in the fact that scientific advice-giving committees are generally called upon to fulfill their most important role just at the point when scientific knowledge is at its least certain; if we were all certain about some piece of science and technology, then there would be no need for any committee structure and we would just do what was natural, like putting insulation on high-voltage wires in the home. The paradox is reinforced, according the authors, by the fact that the prestige of science has rarely been so low. They say, “The aim of this book is to contribute to a theory of scientific advising in which the paradox is resolved.” (p 1)

It would all be so simple if, as under what they call the “standard model,” there was science on the one hand and policy on the other. Scientists would provide the information, and policy makers would use it. For example, the scientists might provide information on the risk/benefit balance of the introduction of, say, a new drug, and the politicians would choose the acceptable balance for society.

The authors explain why things are always much more complicated than this. For example, it is often hard to be clear about where science ends and policy begins. It all depends on what you mean by science. As they cleverly point out, what we mean by the outcome of a scientific deliberation is partly determined by how the Gezondheidsraad goes about selecting the people it will consult: the Gezondheidsraad is the legitimator for what counts as a scientific deliberation, not just a doer of scientific deliberation. This kind of point is illustrated through close observation of a series of passages of decision-making where the authors where present during the various stages and conducted interviews with the principal parties.

Rather than give an exposition of one of their cases, it may be more instructive for those unfamiliar with this kind of thinking to revisit a recent and well-publicised case in the United Kingdom. At the end of October 2009, Professor David Nutt, head of the Advisory Council on the Misuse of Drugs, was sacked by the British Home Secretary. Nutt had publicly criticised the government for reclassifying cannabis from Class C (less dangerous) to Class B (more dangerous and carrying heavier penalties for possession and dealing) in contradistinction to his committee's recommendations. He said that his sacking was a “serious challenge to the value of science in relation to the government.” Whether Nutt should have spoken out or not, we can agree that if Nutt's brief was to do the “science,” then the government had misused its policy-making powers in classifying the drug in a different way to its appointed committee of experts. They might have said, “we accept the classification of C but we are going to treat cannabis as a special case and apply the sanctions of possession and dealing appropriate to a Class B drug,” but they should not have chosen their own scientific classification. Since I have no idea what went on in Nutt's committees (NC) I am going to speculate, using the case as a thought experiment. If NC considered only the physical harm caused by cannabis then the government was wrong. If NC considered the psychological effects of cannabis—e.g., schizophrenia—then things are a little less certain because these illnesses are hard to diagnose. If NC considered the harm caused by reclassification—the imposition of harsh penalties on small-time dealers with no previous criminal record, the creation of a new hardened criminal class, and the increase in muggings and robberies resulting from an inevitable rise in price of the commodity—then what? Is that policy or science? The way the government handled the advice would, in part, define what was in and what was out of the definition of “science.” And how they did this would, in turn, be a matter of how they were judging public opinion. Likewise, advisory committees will be keeping an eye on what is credible advice at any one time and what danger means (car-driving in the United States kills about 110 ordinary people per day, while space-shuttle flight kills about seven volunteers for dangerous missions every five years, but the latter is considered unacceptably dangerous while the former is not). Thus, the sharp division between science and policy, and indeed politics, cannot be maintained. The authors show how cases like this work out in practice.

The observations are interesting but the lasting contribution of the book is likely to be the classification of scientific problems and their treatment, which is found in the concluding chapter and summarised in Table C.1 on page 160. This table encapsulates the way the authors claim to have resolved the paradox with which they started. It comprises a four-way classification of problems and treatments (exemplified by four aspects of the nano-technology debate). *Simple problems*, where all the necessary evidence is, in principle available, require input only from technical experts drawn from within the usual disciplinary boundaries. *Complex problems* begin to depend on a larger range of unknowns and ill-defined issues. The example the authors give is nanotechnology's contribution to genetic engineering and its effect on farming in developing countries. Here, outside experts should be drawn into the discussion that will begin to have more discursive components along with the technical. *Uncertain problems* have aspects that cannot move from unknown to known by extending current techniques or even by using external expertise. The example the authors give is nano-particles' effect on health. Here, there is a role for the precautionary principle—the notion that precautionary measures should be taken even in the face of scientific uncertainty if a given action might cause potentially irreversible threats to public health or environment—and, given the unknowns, stakeholders as well as experts must be drawn in to decide on acceptable balances of risk. *Ambiguous problems* depend clearly on definition and socially sanctioned value judgements. The authors exemplify nano-technology's potential contribution to improving the brain's powers, which might be attractive to some though thought of as “unnatural” or even blasphemous by others. Here, the general public has a role to play from the outset.

This kind of approach is extremely refreshing after a decade or so of attempts to address society's problems with technology by proposing solutions that consist solely of populist demands for “more public participation.” Bijker et al. have realised that science will dissolve away under the assault of more and more lay participation in technological judgments. The classification seems about right, especially as it keeps participants out except where they are needed and thus safeguards the idea of the scientific and technological expert. I cannot forbear from saying, however, that Collins and Evans's (2007) [1] *Rethinking Expertise* contains a classification of expertise would help decide just who belongs in the categories of disciplinary and outside expert. *Rethinking Expertise*, of which the author of this review is a co-author, develops a “Periodic Table of Expertises” that turns on how much of the “tacit knowledge” belonging to a technical domain has been acquired by the expert (or non-expert). Certain old classes of expert are excluded and certain new ones legitimated.

One of the recommendations of the authors is that the Gezondheidsraad continue to hold its deliberations in private. This is likely to surprise some of the book's more “democracy-minded” readers. The authors' justification is that scientific arguments should be managed in a way that allows everyone to speak freely without fear of premature politicization of a scientific opinion. The scientific advice, when it finally emerges, has to appear consensual or it will no longer look like scientific advice. Everyone knows, or should know by now, that scientific consensus is born out of disagreement but science isn't quite science while the disagreement is going on [2]. (This does not prevent the possibility of consensus over how little is fully understood.)

Here, the authors might risk being misread: what they are saying is that the debates should not be watched in real time but we should also bear in mind that if the idea of “discussions in private” were interpreted too narrowly, then we would not have had this study or this book to reassure us that things were going along OK. “Private” debates should be monitored by *representatives* of the public from time-to-time even if everyone agrees that what was said cannot be made fully public until after some delay.[Fig pbio-1000274-g001]

**Figure pbio-1000274-g001:**
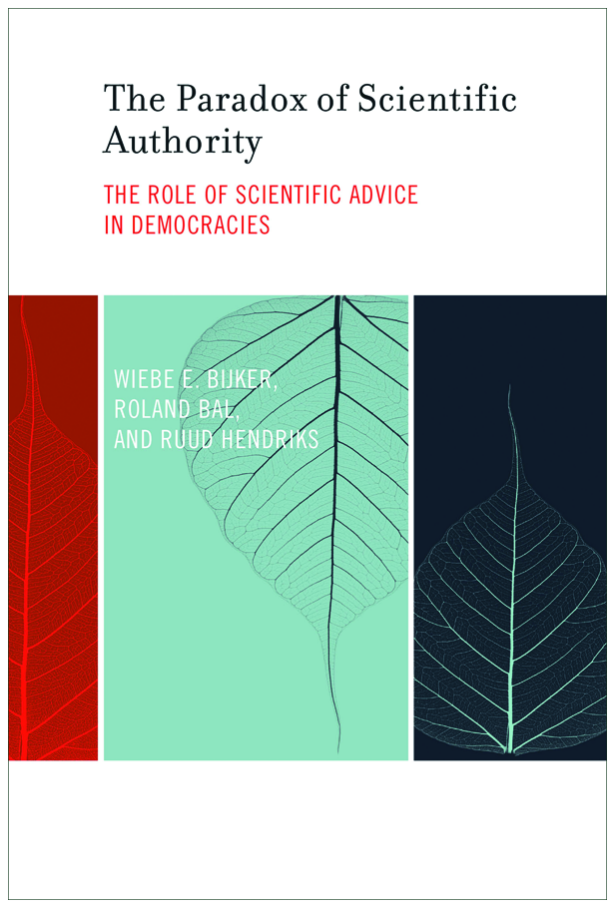
Bijker, Wiebe E, Roland Bal, and Ruud Hendriks (2009) The paradox of scientific authority: the role of scientific advice in democracies. Cambridge, MA: MIT Press. 232 p. ISBN (hardcover): 978-0-262-02658-1 US$32.00.
